# Use of palliative radiotherapy among patients with metastatic non-small-cell lung cancer in Puerto Rico

**DOI:** 10.1186/s12904-021-00819-x

**Published:** 2021-08-13

**Authors:** Valerie Quiñones-Avila, Karen J. Ortiz-Ortiz, Ruth Ríos-Motta, Heriberto Marín-Centeno, Guillermo Tortolero-Luna

**Affiliations:** 1 Department of Health Services Administration, Graduate School of Public Health, Medical Sciences Campus, University of Puerto Rico, P.O. Box 363027, San Juan, 00936-3027 Puerto Rico; 2 Division of Cancer Control and Population Sciences, University of Puerto Rico Comprehensive Cancer Center, San Juan, Puerto Rico; 3 Puerto Rico Central Cancer Registry, University of Puerto Rico, Comprehensive Cancer Center, San Juan, Puerto Rico

**Keywords:** Use of palliative radiotherapy, Non-small cell lung cancer, Puerto Rico

## Abstract

**Background:**

Palliative radiotherapy (RT) represents an important treatment opportunity for improving the quality of life in metastatic non-small cell lung cancer (NSCLC) patients through the management of symptoms within the course of the illness. The aim of the study is to determine the proportion of patients who had palliative RT within 12 months of diagnosis and evaluate the factors associated with it.

**Methods:**

A retrospective cohort study was performed using secondary data analysis from 2009 to 2015 from the Puerto Rico Central Cancer Registry–Health Insurance Linkage Database (PRCCR-HILD). A logistic regression model was used to examine factors associated with palliative RT.

**Results:**

Among the 929 patients identified with metastatic NSCLC, 33.80% received palliative RT within the first year after diagnosis. After adjusting for other covariates, receipt of chemotherapy (OR_Adj_ = 3.90; 95% CI = 2.91–5.45; *P* < 0.001) and presence of symptoms (OR_Adj_ = 1.41; 95% CI =1.00–1.98; *P* = 0.045) were associated with increased odds of palliative RT use. Although marginally significant, patients with private health insurance had increased odds of palliative RT use (OR_Adj_ = 1.50; 95% CI = 0.98–2.29; *P* = 0.061) when compared to beneficiaries of Medicaid, after adjusting by other covariates.

**Conclusions:**

The results of this study reveal concerning underuse of palliative RT among patients with metastatic NSCLC in Puerto Rico. Additional research is necessary to further understand the barriers to using palliative RT on the island.

## Background

Lung cancer is the third most common cancer among males and the fifth most common cancer among females in the Commonwealth of Puerto Rico, an unincorporated territory of the United States [1]. It also ranked as the third leading cause of cancer mortality in both men and women in the island [1]. Although lung cancer incidence and mortality rates are lower in Puerto Rico than in the United States, it remains as significant public health problem. Since most lung cancer patients are diagnosed at an advanced stage of the disease, these patients experience more signs of symptom distress than patients diagnosed with other types of cancer [2]. Symptoms such as pain are frequently followed by the worsening of other symptoms and may affect quality of life [3].

The relief and management of symptoms in lung cancer patients is frequently achieved through the early integration of palliative radiation therapy (RT), particularly for patients with advanced or metastatic non-small cell lung cancer (NSCLC) [3–5]. Palliative RT is used to ultimately improve the quality of life for these patients by treating focal symptoms arising from the primary or metastatic tumor [5], restoring function, preventing the progression of the disease in the treated area or relieving suffering [6]. However, despite the potential benefits of palliative RT, significant variability exists in the integration of this treatment into the healthcare delivery for NSCLC patients worldwide [7]. Variations in treatment may result in worse health outcomes, higher social costs and reduced cost-effectiveness [8].

In Puerto Rico, little is known about the use of palliative care services, such as palliative RT, for NSCLC patients. Also, the lack of training, protocols, and palliative care teams and specialists [9] may exacerbate the gap between the need for palliative care services and availability of these services in the island. This is of great concern because lack of appropriate and timely use of oncologic health services may contribute to the observed differences in cancer-related health outcomes and survival [7, 10]. Therefore, understanding the barriers to standards of care is essential for addressing the disparities in the burden associated with symptom distress experienced by the NSCLC population, which can ultimately result in a low quality of life for these patients [11]. As such, this study aims to characterize the use of palliative RT among metastatic NSCLC patients in Puerto Rico. Information about the utilization profile of palliative RT care in Puerto Rico will help identify barriers to comprehensive oncologic patient care and allow for the development of health interventions to improve care delivery.

## Methods

### Data source

The source of information for this study was the Puerto Rico Central Cancer Registry-Health Insurance Linkage Database (PRCCR-HILD). The PRCCR has been part of the United States National Program of Cancer Registries (NPCR) since 1997. The Surveillance, Epidemiology, and End Results (SEER) Program and the North American Association of Central Cancer Registries (NAACCR) standards are used for coding data. In the most recent NPCR audit, the PRCCR complied with all the criteria, including the completeness of case ascertainment (> 99.0%), comparable to the United States median (99.95%) [12].

As one of the main surveillance systems in the island, the PRCCR monitors all cancer diagnoses obtained from health facilities in Puerto Rico that diagnose or treat cancer patients, such as hospitals, outpatient clinics, pathology laboratories and RT/chemotherapy sites. Demographic characteristics, date of cancer diagnosis, anatomic cancer site, histology type, method of diagnosis, stage of disease at diagnosis, therapy and follow-up status, and cause of death are included in the type of information collected. Data in the PRCCR files is linked to the insurance claims files in order to constitute the PRCCR-HILD. The process of linking claims from the health insurance databases is performed using a deterministic match similar to the one used by SEER-Medicare [13]. All data is de-identified so that the health information is not linked to individual patients. Insurance claims files includes data from Medicare, Medicaid and private health insurers.

The health-care system in Puerto Rico, like that in the United States, has a combination of private and public insurers. In Puerto Rico, nearly 92% of the population is insured and most residents have Medicaid or Medicare (60.0%) [14]. Medicaid is a health insurance program funded by federal and state resources that provides coverage to low-income individuals and families who fit into an eligibility group that is recognized by federal and state law [15] Each state or territory manages the Medicaid program; hence the eligibility requirements can vary from jurisdiction to jurisdiction. In Puerto Rico, Medicaid has a special coverage that includes cancer to help manage these conditions [16]. Nearly half of the Puerto Rico population has Medicaid (46%) [14]. Meanwhile, Medicare is a federally funded national health insurance program that primarily provides coverage for individuals over the age of 65, regardless of their income, and certain individuals under the age of 65 who may qualify due to a permanent disability or other specific circumstance [15]. Additionally, individuals with low income and over the age of 65 or disabled are “dual eligible” since they qualify for both Medicaid and Medicare. Private health insurance, including employer-sponsored insurance and plans directly purchased from an insurer, have accounted for 31.5% of the Puerto Rico population [14]. Likewise, there is other health insurance coverage for the military service members, which receive health care services through special programs such as the Department of Veterans Affairs and TRICARE [15].

Most health insurance plans cover the essential services like outpatient and hospital visits, emergency room visits, and medical devices. Depending on the health insurance type, the out-of-pocket costs vary. Upon providing the service, providers usually bill the insurer, and the patient pays the out-of-pocket. Palliative care, including palliative RT, is billed like any other medical specialty. Many private insurance companies will cover all or part of palliative care. Medicare covers RT costs, but the patient may still be responsible for some out-of-pocket expenses, which varies according to the type of coverage (Part A, B, C, D) [17]. Medicaid coverage varies by state. Palliative care that falls within standard benefits, such as doctor visits and medications, is covered by Medicaid, which is handled by the states according to federal criteria [18].

### Study cohort

**Fig. 1:**
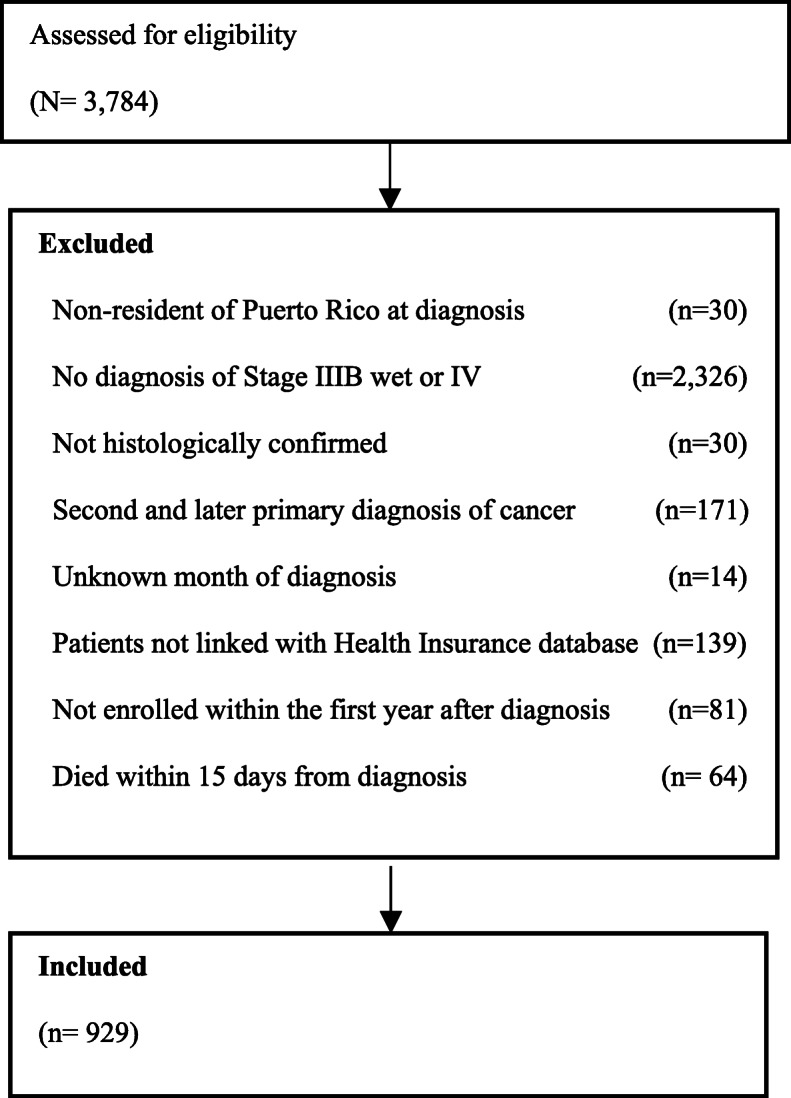
Cohort Selection

This study included patients aged 21 years or older in the PRCCR-HILD with a primary diagnosis and histologic confirmation of metastatic NSCLC (defined as stage III-B with malignant effusion (wet IIIB) or stage IV NSCLC [19] between January 2009 and December 2015. Only residents of Puerto Rico at diagnosis were included. NSCLC was required to be pathologically confirmed and diagnosed prior to death as the primary cancer diagnosis. To ensure we had adequate claims information for our analyses, patients with incomplete claims data regarding cancer diagnosis (cancer type and cancer stage), with multiple primary diagnoses, with missing diagnosis date, without claims data after the primary diagnosis, without enrollment data during the first year after diagnosis, or with a death certificate within 15 days from diagnosis were excluded (Fig. 1).

### Study variables

The variables of interest for this study were identified via data in the PRCCR-HILD as well as insurance claims codes for the Current Procedural Terminology (CPT), Healthcare Common Procedure Coding System (HCPCS), and the International Classification of Diseases for Oncology 3th (ICD-O-3). The patient’s diagnosis was identified using the ICD-O-3 codes for metastatic NSCLC site codes C340-C349, excluding small cell lung cancer (histology codes: 80413, 80,423, 80,433, 80,443, and 80,453), lymphomas, and sarcomas.

Our primary outcome variable, use of palliative RT, was defined by a combination of patient cohort (metastatic NSCLC primary diagnosis) and patient claims using HCPCS, CPT, and ICD codes for radiation courses occurring within 1 year of diagnosis. Codes used for identifying receipt of palliative RT included stereotactic RT, conventional radiation treatment delivery, conventional IMRT delivery, high-energy neutron radiation, and proton beam radiation treatment delivery, as defined in similar studies [20, 21]. Previous work with surveillance registry has also considered that the RT for patients with advanced NSCLC is being provided with palliative intent as a standard of care [20].

The independent variables were categorized in order to assess for nonlinear trends. Demographic variables included age, sex, and marital status. Health services variables included health insurance type, density of RT centers, and geographic location. Density of RT centers was defined as the proportion of RT center per region of patient residency, as defined by Health Department Coalitions by Health Region [22, 23]. This variable was categorized according to the distribution of the study sample: low (0–2 centers), medium (3–6 centers), high (> 7 centers). Geographic location referred to whether or not the patient’s municipality of residency belonged to a metropolitan area, as defined by the Office of Management and Budget (OMB) [24, 25]. Clinical variables included surgery, chemotherapy, year of diagnosis (2009–2015), symptoms and comorbidity. Surgery and chemotherapy receipt were identified at any time within 1 year of diagnosis. Common symptoms, due to cancer or its treatment, that are commonly treated with palliative RT were identified in the claims data according to literature [3, 4, 26]. This variable was defined as having at least one of the following: malaise and fatigue, generalized pain, dyspnea and respiratory abnormalities, dysphagia, acute pain, cough, hemoptysis, chest pain, brachial plexopathy, or superior cava syndrome. Comorbidity was assessed during 1 year prior diagnosis using the modified Charlson’s comorbidity index described by Klabunde et al. [27, 28].

### Statistics

First, descriptive statistics were used to examine the characteristics of the study population. Then, the proportion of patients who had palliative RT was determined. Univariate associations with palliative RT were determined with two-tailed Pearson chi-squared tests. Univariate logistic regression models were performed in order to determine the unadjusted magnitude of the associations. Then, all covariates, regardless their statistical significance in the univariate models, were included in a multivariate logistic regression model to assess covariates independently associated with the use of palliative RT. Exploratory analysis was conducted to identify interactions between the independent variables. These would be accounted for in the multivariate model if considered appropriate. Statistical significance was set at the *P* = 0.05 level. Results were presented as odds ratio (OR) with 95% confidence intervals and *P* values. Statistical analyses were performed using STATA software (Stata Corp., LP. College Station, TX).

## Results

Patient characteristics for the 929 study participants are presented in Table 1**.** The age of the study patients ranged from 28 to 96 years old. The median age was 67. The majority of the patients consisted of males (59.42%) in the 60–74 age category (48.65%), married (54.68%), and with Medicaid as the primary payer (31.22%). A vast majority resided in a Metro region (96.23%), and more than half of the regions had a medium density of RT centers (65.12%). Although most patients did not receive surgery (91.17%), almost half of the patients received chemotherapy (47.47%). Finally, although the majority (66.52%) had no comorbidities, 65.66% experienced pain.

**Table 1:** Baseline Characteristics and Univariate Predictors of Palliative RT Among Patients with NSCLC in Puerto Rico

		Palliative RT		
Characteristic	Total (%)	No n (row %)	Yes n (row %)	*P* value
All patients	929 (100.0)	615 (66.20)	314 (33.80)	
**Age group (years)**				**0.011**
21–59	258 (27.77)	155 (60.08)	103 (39.92)	
60–74	452 (48.65)	300 (66.37)	152 (33.63)	
75+	219 (23.57)	160 (73.06)	59 (26.94)	
**Sex**				0.732
Male	552 (59.42)	363 (65.76)	189 (34.24)	
Female	377 (40.58)	252 (66.84)	125 (33.16)	
**Marital Status**				0.596
Unmarried	396 (42.63)	269 (67.93)	127 (32.07)	
Married	508 (54.68)	329 (64.76)	179 (35.24)	
Unknown	25 (2.69)	17 (68.00)	8 (32.00)	
**Health Insurance**				**< 0.0001**
Medicaid	290 (31.22)	208 (71.72)	82 (28.28)	
Medicare	201 (21.64)	124 (61.69)	77 (38.31)	
Medicare-Medicaid	234 (25.19)	170 (77.65)	64 (27.35)	
Private	204 (21.96)	113 (55.39)	91 (44.61)	
**Density of RT Centers**				0.400
Low (0–2)	71 (7.64)	49 (69.01)	22 (30.99)	
Medium (3–6)	605 (65.12)	407 (67.27)	198 (32.73)	
High (7+)	253 (27.23)	159 (62.85)	94 (37.15)	
**Geographic Location**				0.163
Metro Area	894 (96.23)	588 (65.77)	306 (34.23)	
Non-Metro Area	35 (3.77)	27 (77.14)	8 (33.80)	
**Surgery**				0.249
No	847 (91.17)	556 (65.64)	291 (34.36)	
Yes	82 (8.83)	59 (71.95)	23 (28.05)	
**Chemotherapy**				**< 0.0001**
No	488 (52.53)	397 (81.35)	91 (18.65)	
Yes	441 (47.47)	218 (49.43)	223 (50.57)	
**Year of Diagnosis**				
2009	107 (11.52)	70 (65.42)	37 (34.58)	0.997
2010	85 (9.15)	55 (64.71)	30 (35.29)	
2011	121 (13.02)	82 (67.77)	39 (32.23)	
2012	151 (16.25)	102 (67.55)	49 (32.45)	
2013	156 (16.79)	101 (64.74)	55 (35.26)	
2014	148 (15.93)	99 (66.89)	49 (33.11)	
2015	161 (17.33)	106 (65.84)	55 (34.16)	
**Symptoms**				**< 0.0001**
No	319 (34.34)	239 (74.92)	80 (25.08)	
Yes	610 (65.66)	376 (64.64)	234 (38.36)	
**Comorbidity**				0.462
0	618 (66.52)	406 (65.70)	212 (34.30)	
1	153 (16.47)	98 (64.05)	55 (35.95)	
2+	158 (17.01)	111 (70.25)	47 (29.75)	

*Abbreviation: NSCLC* non–small-cell lung cancer, *RT* radiation therapy

Overall, 33.80% of the study participants received palliative RT within one year of diagnosis. Table 1 also lists the distribution of palliative RT use and non-use by patient characteristics. In the bivariate analysis, associations with palliative RT use were seen with age, health insurance, receiving chemotherapy, and symptoms (*p* < 0.05). The use of palliative RT decreased with increasing age categories (39.92% for ages 28–59; 33.63% for ages 60–74, and 26.94% for ages 75+; *P* = 0.011). Meanwhile, it was higher in patients with private health insurance (44.61% vs 28.28, 38.31, 27.35%, respectively; *P* < 0.001); patients receiving chemotherapy (50.57% vs 18.65%; *P* < 0.001); and patients experiencing symptoms (38.36% vs 25.08%; *P* < 0.001) when compared to their respective counterparts.

Results for the univariate logistic regressions were consistent with the Pearson Chi-Squared results (Table 2). Potential interactions between health insurance and covariates were also evaluated. However, the terms of interaction were not statistically significant (*P* = 0.3280). Therefore, all the covariates, regardless of their statistical significance, were included in a multivariate logistic regression model to identify covariates independently associated with the use of palliative RT (Table 2*).* Although age lost its significance, private health insurance remained marginally significant, and both chemotherapy treatment and symptomatology remained statistically significant, after adjusting for other covariates. In the adjusted model, patients treated with chemotherapy were nearly four times more likely to receive palliative RT than their counterparts (OR = 3.90; 95% CI: 2.91, 5.45; *P* < 0.0001). Furthermore, patients with symptoms were 41% more likely to receive palliative RT than patients without symptoms (OR = 1.41; 95% CI: 1.00, 1.98; *P* = 0.045), after adjusting for other covariates. Finally, patients with private health insurance were 50% more likely to receive palliative RT than patients with Medicaid (OR = 1.50, 95% CI: 0.98–2.29; *P* = 0.061).

**Table 2:** Predictors of Palliative RT among Metastatic NSCLC Patients in Puerto Rico

Characteristic	Unadjusted Model		Adjusted Model	
	Odds Ratio (95% CI)	P Value	Odds Ratio (95% CI)	P Value
**Age group (years)**				
21–59	1.00	–	1.00	–
60–74	0.76 (0.56, 1.04)	0.093	0.80 (0.55, 1.16)	0.231
75+	0.55 (0.38, 0.82)	**0.003**	0.71 (0.44, 1.17)	0.182
**Sex**				
Male	1.00	–	1.00	–
Female	0.95 (0.72, 1.25)	0.732	0.87 (0.64, 1.19)	0.408
**Marital Status**				
Unmarried	1.00	–	1.00	–
Married	1.15 (0.87, 1.52)	0.318	0.91 (0.66, 1.26)	0.571
Unknown	0.99 (0.42, 2.37)	0.994	0.87 (0.34, 2.23)	0.779
**Health Insurance**				
Medicaid	1.00	–	1.00	–
Medicare	1.58 (1.07, 2.31)	**0.020**	1.47 (0.92, 2.37)	0.109
Medicare-Medicaid	0.95 (0.65, 1.40)	0.814	1.02 (0.64, 1.63)	0.920
Private	2.04 (1.40, 2.98)	**< 0.0001**	1.50 (0.98, 2.29)	**0.061**
**Density of RT Centers**				
Low (0–2)	1.00	–	1.00	–
Medium (3–6)	1.08 (0.64, 1.84)	0.767	1.13 (0.62, 2.04)	0.685
High (7+)	1.32 (0.75, 2.31)	0.339	1.31 (0.70, 2.48)	0.394
**Geographic Location**				
Metro	1.00	–	1.00	–
Nonmetro	0.57	0.168	0.70 (0.29, 1.66)	0.416
**Surgery**				
No	1.00	–	1.00	–
Yes	0.74 (0.45, 1.23)	0.250	0.66 (0.39, 1.14)	0.137
**Chemotherapy**				
No	1.00	–	1.00	–
Yes	4.46 (3.32, 5.99)	**< 0.0001**	3.90 (2.91, 5.45)	**< 0.0001**
**Year of Diagnosis**				
2009	1.00		1.00	–
2010	1.03 (0.57, 1.87)	0.918	1.03 (0.54, 1.97)	0.932
2011	0.89 (0.52, 1.56)	0.707	0.98 (0.54,1,79)	0.959
2012	0.91 (0.54, 1.54)	0.721	0.88 (0.50, 1.56)	0.659
2013	1.03 (0.61, 1.73)	0.910	0.96 (0.54, 1.68)	0.879
2014	0.94 (0.55, 1.58)	0.806	1.04 (0.59, 1.85)	0.870
2015	0.98 (0.58, 1.64)	0.944	1.19 (0.68, 2.10)	0.545
**Symptoms**				
No	1.00	–	1.00	–
Yes	1.86 (1.38, 2.51)	**< 0.0001**	1.41 (1.00, 1.98)	**0.045**
**Comorbidity**				
0	1.00		1.00	–
1	1.07 (0.74, 1.55)	0.702	1.02 (0.68, 1.55)	0.919
2+	0.81 (0.55, 1.18)	0.279	0.86 (0.55, 1.33)	0.483

*Abbreviations: CI*, confidence interval, *NSCLC* non–small-cell lung cancer, *OR* odds ratio, *RT* radiation therapy

## Discussion

Our findings suggest a lower use of palliative RT among patients with metastatic NSCLC in Puerto Rico (33.80%) when compared to patients in the United States (50% or greater) [21, 29–31] and patients in developing countries (28% median rate, though not metastatic lung cancer specific) [32]. The reasons for the underuse of palliative RT identified in this study remain to be determined. They could be multifactorial, as several population-based studies of clinical practice patterns in US patients have found that disparities in this type of care are influenced by sociodemographic factors (such as age), clinical factors (such as chemotherapy treatment or stage of cancer), and financial structures (such as health insurance reimbursement structure) [29, 33, 34].

Additionally, the available medical services in Puerto Rico might be failing to refer palliative RT treatment due to provider stigma, unawareness, lack of staff, or even more robust clinical guidelines [35]. Training protocol inadequacies, decreased comfort level, and decreased access to experts in palliative care may exacerbate the gap between the need for palliative care services and the usage of these services in the island [35]. Other factors, including cultural beliefs, and the existence or lack of social support could also affect the ability to receive recommended care [10].

The low rate of palliative RT among the metastatic NSCLC population Puerto Rico is concerning, given the value of this treatment in improving the quality of life. By drawing attention to the underuse of guideline-concordant care for metastatic NSCLC in Puerto Rico, this study has the potential to improve oncologic treatment in the island. This is the first step in assessing the unmet need for palliative RT in terms of those who receive it vs those who, despite having the indication, do not have access to it [32]. A more detailed picture of palliative care that currently exists in cancer centers in Puerto Rico can help identify deficiencies and barriers to comprehensive cancer care and allow development of health interventions to improve care delivery.

This study also demonstrates disparities in palliative RT use by clinical and demographic characteristics. Receipt of chemotherapy was associated with increased odds of palliative RT use among NSCLC patients, after adjusting for other covariates. Meanwhile, decreased odds of palliative RT use was associated with increasing age categories in the unadjusted model, but lost its significance after adjusting for other covariates. These findings support prior work considering receipt of chemotherapy and younger age at diagnosis as proxies for higher performance status of patients who may withstand the rigors of more intensive treatment, such as palliative RT [19, 29, 33, 36]. Also, an age disparity with palliative RT use has been consistently found among older patients with advanced cancer [19, 21, 29, 37], suggesting a gap between quality of cancer care and the needs of a population that might benefit from it. Reasons for this disparity could potentially include higher comorbidity, shorter survival, less disposition, and lack of transportation to receive more intensive cancer care among the elderly, which could impact both the patient’s and doctor’s decision to use palliative RT [37].

Moreover, in this study, patients with symptoms were 41% more likely to receive palliative RT than patients without symptoms, after adjusting for other covariates. However, the majority of patients experiencing symptoms (64.64%) did not end up receiving palliative RT. Perhaps the findings from this study might indicate a disparity in access to palliative RT among patients with symptomology due perhaps to patient refusal of treatment [30]. Previous studies have included symptomology as a covariate for measuring effectiveness of palliative RT [38, 39], rather than as a predictor for palliative RT use.

Although marginally significant, after adjusting for other covariates, patients with private health insurance were 50% more likely to receive palliative RT than patients on Medicaid. One reason for this disparity, well known in literature, is that lower socioeconomic status could affect a patient’s ability to pay deductibles or copayments, which could affect his or her willingness to receive palliative RT [21]. Another reason for this difference may be that patients with a lower socioeconomic status have less access to transportation, which could also limit their access to the radiation center [21, 40]. Lastly, it has been observed that palliative RT can differ according to the reimbursement structure [29]. For Puerto Rico, this indicates that a change of the financial incentives for palliative RT care may be required because reimbursement-related financial incentives may be promoting the under-reporting of palliative RT care in the island (33.80%), which could lead to biased assessments of the utilization and expenditure of care.

There are several limitations in this study. For example, we could not assess important information on how the palliative RT was delivered, such as site of radiation, number of fractions, physician intent, and physician preferences, because the PRCCR-HILD does not collect such information. Lastly, mortality might have affected the study’s internal validity because participants may have died during the period of interest for this study, due to the metastatic stage of our study population. To address this source of bias, we excluded those patients that died within 15 days of diagnosis (*n* = 64). Finally, this study may be potentially relevant only to the cancer community in Puerto Rico and not necessarily generalizable to other communities in the United States or elsewhere.

## Conclusion

Our findings have implications for the oncologic care of patients with metastatic NSCLC in Puerto Rico.. The results of this study reveal a potential underuse of palliative RT among patients with metastatic NSCLC in Puerto Rico. Additional research is necessary to further understand the barriers to using palliative RT on the island. Additionally, the identified disparities in chemotherapy use implies the need to further understand how palliative RT is delivered in conjunction with chemotherapy in Puerto Rico, and the development of updated guidelines to address the clinical implications. Finally, disparities in symptomatology imply that although the presence of symptoms is a facilitator for palliative RT use, early integration of palliative RT is key to effectively manage those symptoms.

## Data Availability

The data that support the findings of this study are available from PRCCR but restrictions apply to the availability of these data, which were used under license for the current study, and so are not publicly available. Data are however available from the authors upon reasonable request and with permission of PRCCR.

## Abbreviations

CDC: Centers of Disease Control
CI: confidence interval
CPT: Current Procedural Terminology
HCPCS: Healthcare Common Procedure Coding System
ICD-O: International Classification of Diseases for Oncology
NAACCR: North American Association of Central Cancer Registries
NPCR: National Program of Cancer Registries
NSCLC: non-small cell lung cancer
OR: Odds Ratio
OR_Adj_: Odds Ratio (Adjuasted)
PR: Puerto Rico
PRCCR: Puerto Rico Central Cancer Registry
PRCCR-HILD: Puerto Rico Central Cancer Registry–Health Insurance Linkage Database
RT: Radiotherapy
SEER: Surveillance, Epidemiology, and End Results
